# How do healthcare providers use national audit data for improvement?

**DOI:** 10.1186/s12913-023-09334-6

**Published:** 2023-04-24

**Authors:** Grazia Antonacci, Julie Whitney, Matthew Harris, Julie E. Reed

**Affiliations:** 1 Department of Primary Care and Public Health, Imperial College London, National Institute of Health Research (NIHR) Applied Research Collaboration (ARC) Northwest London, London, UK; 2 Business School, Centre for Health Economics and Policy Innovation (CHEPI), Imperial College London, London, UK; 3 Department of Physiotherapy, King’s College London, London, UK; 4 Department of Primary Care and Public Health, Imperial College London, South Kensington, UK; 5 School of Health and Welfare, Halmstad University, Halmstad, Sweden; 6 Julie Reed Consultancy Ltd, London, UK

**Keywords:** National clinical audit, Audit, Feedback, Health care improvement, Inpatient falls, Quality improvement

## Abstract

**Background:**

Substantial resources are invested by Health Departments worldwide in introducing National Clinical Audits (NCAs). Yet, there is variable evidence on the NCAs’ effectiveness and little is known on factors underlying the successful use of NCAs to improve local practice. This study will focus on a single NCA (the National Audit of Inpatient Falls -NAIF 2017) to explore: (i) participants’ perspectives on the NCA reports, local feedback characteristics and actions undertaken following the feedback underpinning the effective use of the NCA feedback to improve local practice; (ii) reported changes in local practice following the NCA feedback in England and Wales.

**Methods:**

Front-line staff perspectives were gathered through interviews. An inductive qualitative approach was used. Eighteen participants were purposefully sampled from 7 of the 85 participating hospitals in England and Wales. Analysis was guided by constant comparative techniques.

**Results:**

Regarding the NAIF annual report, interviewees valued performance benchmarking with other hospitals, the use of visual representations and the inclusion of case studies and recommendations. Participants stated that feedback should target front-line healthcare professionals, be straightforward and focused, and be delivered through an encouraging and honest discussion. Interviewees highlighted the value of using other relevant data sources alongside NAIF feedback and the importance of continuous data monitoring. Participants reported that engagement of front-line staff in the NAIF and following improvement activities was critical. Leadership, ownership, management support and communication at different organisational levels were perceived as enablers, while staffing level and turnover, and poor quality improvement (QI) skills, were perceived as barriers to improvement. Reported changes in practice included increased awareness and attention to patient safety issues and greater involvement of patients and staff in falls prevention activities.

**Conclusions:**

There is scope to improve the use of NCAs by front-line staff. NCAs should not be seen as isolated interventions but should be fully embedded and integrated into the QI strategic and operational plans of NHS trusts. The use of NCAs could be optimised, but knowledge of them is poor and distributed unevenly across different disciplines. More research is needed to provide guidance on key elements to consider throughout the whole improvement process at different organisational levels.

**Supplementary Information:**

The online version contains supplementary material available at 10.1186/s12913-023-09334-6.

## Background

From 1990 onwards, National Health Service (NHS) England directed substantial resources to the development and introduction of clinical audit throughout the NHS.[1] Although audit has become an accepted part of good clinical practice, there are still doubts about whether it is achieving its goal of ensuring high quality care.[2–10] Similar uncertainties have been expressed in the USA, where audit has been a routine requirement since 1974.[11, 12].

UK National Clinical Audits (NCAs) are large-scale datasets using information collected locally by clinicians and designed to improve patient outcomes across a range of medical, surgical, and mental health conditions. They are distinguished from other forms of clinical audits by their national coverage and hence the ability to benchmark clinical and organizational performances.[13, 14].

NCAs represent a rich resource of data available to a wide range of stakeholders (healthcare professionals, managers, policy makers, patients, researchers) to drive improvement in patient outcome.[13–17] They were first introduced in the UK in 1990s and there are now more than 50.[4] Most UK NCAs are overseen by the Healthcare Quality Improvement Partnership (HQIP), through the National Clinical Audit and Patient Outcomes Programme (NCAPOP).[13, 15, 18] NCAPOP collects outcome or process data from local healthcare providers, analyses these data centrally and provides feedback locally.[13] The primary output of NCAs is benchmarked reports on the performance of local NHS trusts.[17, 19] These reports can be used by individuals, clinical teams and organization to assess their performance over time or against evidence-based standards with the expectation to prompt local service improvement.[13, 19].

However, there is variable evidence on the effectiveness of NCAs and on the extent to which healthcare providers engage with NCAs feedback to drive improvement.[2, 13, 18–20] Relatively few studies have explored how NCAs outputs are used locally to improve clinical practice and little is known on how best to design NCAs to achieve this aim.[11–13, 18, 19].

A recent study contributed to the understanding of the variation in the use and impact of UK NCAs, confirming the relevance of this area of enquiry both for literature and practice.[19] While this study focused on why, how and in what contexts NCAs are used to stimulate quality improvement,(QI) little is known on how NCAs processes can be improved to increase their impact on local clinical practice. Limited evidence is available as to the important characteristics of Audit and Feedback (A&F) processes from a clinician’s perspective [21, 22] and no study to our knowledge has investigated how the use of NCA as a QI tool can be improved from the perspective of local front-line staff engaged with different roles in the A&F process.

The variable evidence on the effectiveness of NCAs and the limited knowledge about factors underlying their successful use for improvement is mirrored in the wider A&F literature.[23–30].

The National Institute for Clinical Excellence (NICE) [31] sets out broad practical considerations for 5 stages of the A&F process (preparing the audit, selecting criteria, measuring performance, making improvements, and sustaining improvement) without describing in detail the way in which A&F should be conducted.[31].

Knowledge distributed across disciplines exists to inform more effective A&F interventions,[3, 32–35] but evidence on specific features of the A&F process underling its effectiveness as a QI intervention is scarce in the healthcare literature.[2, 36].

The 2012 Cochrane review of A&F identified a list of factors that could explain some variation in the effectiveness of A&F: feedback format (verbal and written), source (colleague or supervisor), frequency, improvement strategies (goal setting and action planning) and baseline performance.[24] However these factors don’t represent an exhaustive list of all elements that need to be considered when designing an A&F intervention.[25, 37].

Drawing on the 5 modifiable elements of A&F design identified by the Improved Clinical Effectiveness through Behavioural Research Group (ICEBeRG),[38] Colquhoun et al. developed a list of 17 modifiable A&F design elements which are applicable to most A&F interventions. These elements are organised into the following 6 categories: to whom the A&F was delivered; what audited information was delivered, when it was delivered (what was the lag time between practice and feedback), why it was provided (what was the rationale for using A&F), how it was delivered and how much (the number of feedback instances delivered).[37] Hysong conducted a meta-analytic study, revealing that A&F effectiveness is improved when feedback is delivered with specific suggestions for improvement, in writing and frequently [36]; while Brehaut and colleagues,[39] identified 15 suggestions that are likely to improve the effectiveness of feedback across a range of contexts. These include the nature of the desired action, the nature of the data available for feedback, feedback display and delivering the feedback intervention.

Although A&F literature provides useful insights about A&F features which could improve its effectiveness, they focus more on technical aspects of A&F design, than on aspects such as organisational culture, management support, QI skills, and other social aspects which are key to the success of any QI intervention according to Improvement and Implementation Science literature and Behavior Change literature.[3, 40–46].

Many theories developed across different disciplines (e.g. industrial and organisational psychology) are contributing A&F healthcare literature to further explain mechanisms underlying A&F effectiveness as QI intervention.[3, 32] For example, behaviour change theories and organisational theories suggest that A&F interventions support QI as they make providers aware of their suboptimal performances [47] or focus on effect modifiers with respect to QI (e.g. organisational culture and support), and the ‘actionability’ of feedback reports.[20, 48] Empirical evidence from non-healthcare literature also suggests that goal setting can increase the effectiveness of feedback [49] and endorses the value of action-plans to improve feedback effectiveness.[50] Kluger and De Nisi [51] developed a Feedback Intervention Theory (FIT) suggesting that behaviour is regulated by comparing feedback to hierarchical organised goals and standards, and that only gaps that receive attention have the potential for change. They also identify 3 factors determining how effectively this attentional shift occurs: (i) characteristic of the feedback (content, format and frequency); (ii) the nature of the task performed, and (iii) situational and personality variables.

However, despite the increasing number of studies attempting to explain the reasons behind the variation in NCAs and more in general A&F’s effectiveness, available evidence is fragmented across different disciplines.[24, 25, 52, 53] This can be attributed to different factors, including the heterogeneity and complexity of provider behaviour change interventions and the poor reporting of interventions in primary studies [24, 37] compounded by the limited use of theory in the design, implementation, evaluation and reporting of A&F interventions.[3, 37, 54–58].

Given that significant resources, including clinicians’ time are increasingly invested in NCAs, greater research effort needs to be devoted to understand and improve the consistency and magnitude of the NCAs’ effects.[2, 13, 21, 24, 28, 33, 36] This research should identify underlying mechanisms through which feedback is effective and understand ingredients required to produce the most desirable effects of A&F as a QI tool.[2, 33] It therefore should consider human, social and organisational factors, alongside technical factors of A&F.

In this study we aim to advance current understanding on the reasons behind the variation in the extent to which NCAs feedback stimulates QI within single healthcare organisations.

We explore front line staff perspectives on a single NCA (the National Audit of Inpatient Falls - NAIF 2017, one of the NCAs overseen by HQIP) [59] to investigate: (i) what NCA report features, local feedback characteristics and actions undertaken following the feedback underlie the effective use of NCA feedback to improve local practice; (ii) reported changes in local practice following the NCA feedback.

## Methods

Inductive qualitative methods and an iterative study design characterised by cycles of simultaneous data collection and analysis have been used to understand front-line staff attitudes and experiences.[60].

This study explores the perspectives of front-line healthcare professionals in the context of the NAIF programme in different hospitals in England and Wales.

The Consolidated criteria for reporting qualitative research (COREQ) [61] has been used as the reporting guideline for this qualitative study [see Additional file 1].

### Setting

NAIF is commissioned by the HQIP and is managed by the Royal College of Physicians (RCP) as part of the Falls and Fragility Fracture Audit Programme (FFFAP), alongside the Fracture Liaison Service Database (FLS-DB) and the National Hip Fracture Database (NHFD).[59] NAIF aims at improving fall risk reduction practice for inpatients. Falls are the most frequently reported incident affecting hospital inpatients, with 247,000 falls occurring in inpatient settings each year in England alone.[62] Falls among older patients are more likely to result in a severe harm.[62] For example for older people, hip fracture is the commonest reason for emergency surgery and injury related death.[63] Moreover inpatient falls are costly and even where life-changing injuries are not sustained, such events lead to increased length of stay, loss of confidence, restriction of physical activity, functional impairment, diminished independence and an increased risk of further falls. All of which affect patients’ quality of life.

The evidence as to the best way to prevent inpatient falls is not yet conclusive.[64] NICE clinical guideline calls for a multi-factorial falls risk assessment (MFRA) for all inpatients aged over 65 (and in those aged 50–64 who are clinically judged to be at risk), followed by clinical interventions tailored to address identified risk factors.[65] This is a complex task requiring a multidisciplinary team approach and individually tailored interventions.

NAIF audits the delivery and quality of care for patients over 65 who fall and sustain a fracture of the hip or thigh bone in all eligible NHS trusts/health boards in England and Wales. The first NAIF was launched in 2015 and another round following the same approach was conducted in 2017.[66] These were patient level ‘snapshot audits’ of fall prevention activity in acute hospitals in England and Wales. Since then, NAIF became a continuous audit. This study focuses on NAIF 2015 and 2017 as data collection was conducted in 2018 and 2019. Data were collected from 90% (179/ 198) of hospitals in England and Wales in 2015 and 95% (n = 187/197) in 2017. In both audits, a sample of up to 30 patients (15 consecutively admitted patients over 2 days) aged over 65 were assessed for each hospital, with a total of over 5,000 inpatients involved in each audit.

Data collected included evidence of components of MFRA and linked interventions from clinical notes and bedside observations.

**Fig. 1:**
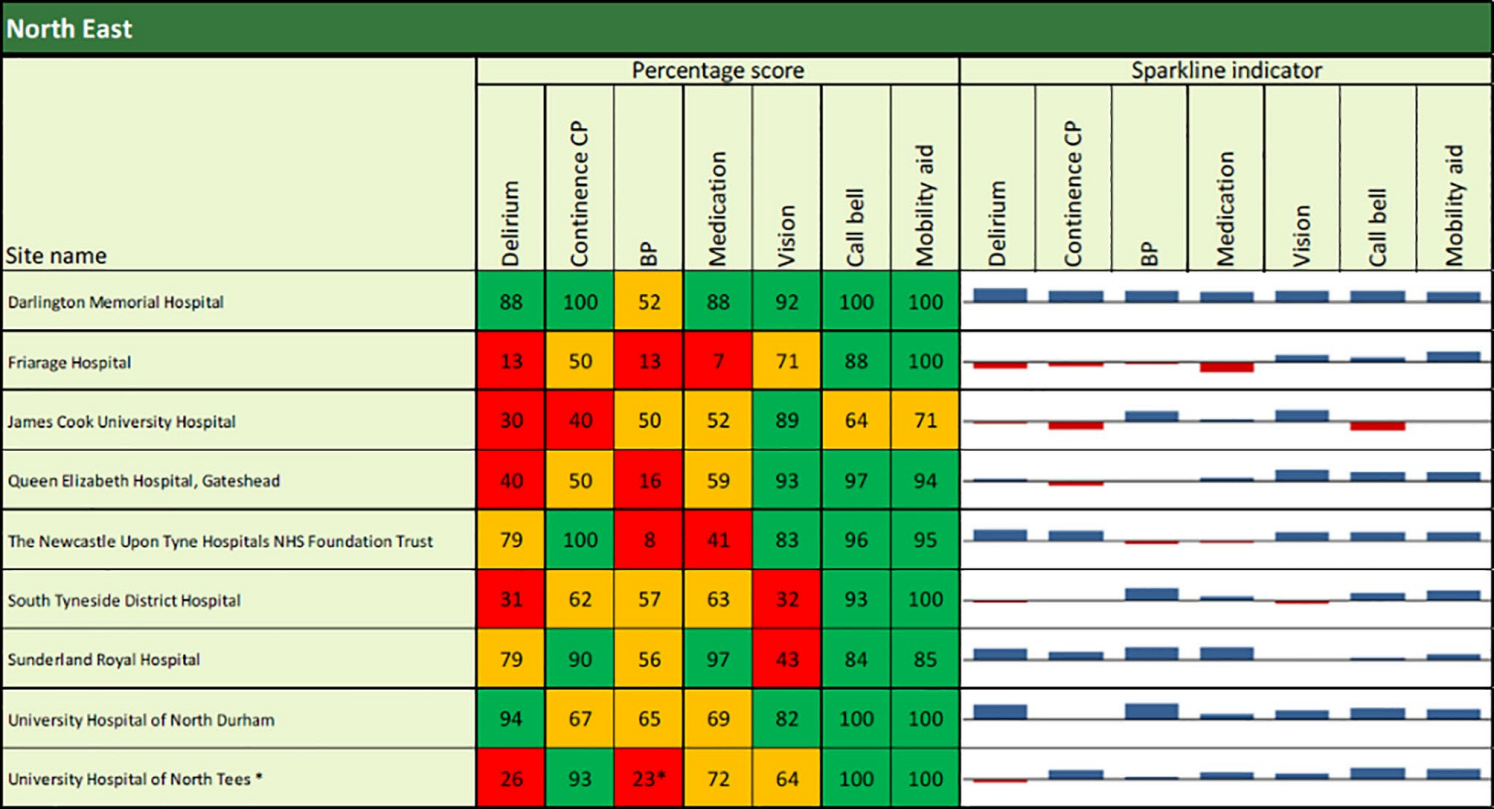
Snapshot of site-level results for key indicators as presented in the Annual Report 2017. Cut‐off values: 0–49% (red), 50–79% (amber) and 80–100% (green), to enable organisations to see where they need to concentrate their interventions and action plans. Sparkline indicators calculated using Z scores: blue blocks (areas above the national average); red blocks (areas below the national average); size of the blocks (how far an organisation is away from the mean) [66]

The main output of the NAIF audits 2015 and 2017 were 2 Annual Reports providing general recommendations for managers and clinical teams, some quality improvement case studies and detailed audit results from all individual hospitals. Hospital-level results enable comparison of hospital performance against the guidance standards, alongside a comparison with other hospitals and, for the 2017 report, a comparison with their own performance in 2015. In the reports, results for each of the seven key indicators for each hospital are presented in tables using colour coded and sparkline indicators. (Fig. 1)

For both audits the data collection was followed by a further period of dissemination and support for hospital’s use of the data. Improvement activities were supported by the RCP in the form of workshops and the development of tools to address areas of poor performance. However, the second round of data collection in 2017 found little or no difference in the national picture for the Key Performance Indicators (KPIs).[66].

### Ethical considerations

The study was reviewed and approved by the NHS Health Research Authority (Integrated Research Application System -IRAS- project ID 236,594) and has been included in the National Institute for Health and Care Research (NIHR) Clinical Research Network Portfolio. All participants were provided with an information sheet detailing the objectives of the study and their rights as participants. Written informed consent was obtained from each participant prior to their involvement, with participants being informed of their right to decline to take part and/or leave at any time. Participation was voluntarily and confidentiality protected. Participants within each site were identified by the local NAIF clinical leads by peer nomination, prioritised by the research team to maximise the diversity of the roles involved, and recruited through email.

### Participant sampling and recruitment

Participants were recruited by purposively sampling hospitals and interviewees on theoretical grounds. Sampling, data collection and analysis continued until data saturation was reached.

*Hospitals’ sampling*: The hospitals’ sample included seven hospitals from a pool of 85 hospitals in England and Wales joining the 2015 and 2017 NAIF audits which consented to participate. The sampling strategy used aimed to include hospitals with different NAIF performances between 2015 and 2017. The sample included hospitals homogeneous in terms of size but reporting different levels of performance: 3 hospitals registering a performance drop (H1, H2, H3), 2 hospitals that improved their inpatient falls’ performances (H4, H5), 2 hospitals with steady performances (H6, H7) and were randomly selected by the NAIF programme team. Sampling criteria were agreed among the research team and verified by key informants, including NAIF programme team.

*Interviews’ participants sampling*: Sampling from hospital staff was performed to provide a broad range of relevant perspectives and to increase generalizability of findings.[67] Clinical leads at participating trusts were asked by the NAIF programme team to identify NHS staff involved in the NAIF audit process. Participants with different roles in the audit and different backgrounds were selected and recruited for interviews by the research team. (Table 1) Sampling and recruitment were conducted simultaneously in the different hospitals until data saturation.

**Table 1:** Interviewees

Hospital ID	Role	Interviewee Abbreviation
H1	Matron	*H1_Matron*
H1	Consultant	*H1_Consultant*
H1	Sister	*H1_Sister*
H1	Consultant	*H1_Consultant*
H1	Matron	*H1_Matron*
H2	Falls Lead Nurse	*H2_Falls Lead Nurse*
H2	Assistant Director of Nursing	*H2_Assistant Director of Nursing*
H3	Nurse	*H3_Nurse*
H4	Consultant	*H4_Consultant*
H5	Matron	*H5_Matron*
H5	Patient Safety Lead Nurse	*H5_Patient Safety Lead Nurse*
H5	Consultant	*H5_Consultant*
H5	Matron	*H5_Matron*
H5	Consultant	*H5_Consultant*
H6	Falls Lead Nurse	*H6_Falls Lead Nurse*
H6	Practice Development Physiotherapist (supports falls lead nurse)	*H6_Physiotherapist*
H7	Falls Lead Nurse	*H7_Falls Lead Nurse*
H7	Consultant	*H7_Consultant*

### Data collection

Eighteen semi-structured interviews were conducted between December 2018 and April 2019. Interviews took about 45–60 min and were conducted by telephone by a research associate with a PhD in the area of health services research and experience with qualitative research methods (GA, female).

The interview guide was informed by literature [24, 25, 31] and co-developed with NAIF programme team and clinical leads at participating trusts. It was then pilot tested and progressively refined during the study. Before starting the interview, GA briefly introduced herself, the reasons for doing the research and she clarified that she was an independent researcher from Imperial College, not a NAIF team member. She also re-iterated the message that the interview was confidential and anonymised. The themes explored included: participants’ experience with audit and feedback, the way in which NAIF audit and feedback process was conducted within each hospital, its contribution to the reduction of inpatients falls and reported changes in behaviour, key elements that helped the NAIF audit as well as problems and challenges related to its effective use as QI tool [see Additional file 2]. Interviews were audio recorded, anonymised, and transcribed by independent professional transcriptionists. Repeat interviews were not needed. Transcripts were not returned to participants, given the difficulty to engage with busy healthcare professionals.

### Data analysis

Qualitative data analysis was guided by constant comparative techniques.[68] NVivo software was used to analyse the interviews. To have a more objective analysis of the collected qualitative data, the first stage of analysis of interview data were blinded with respect to the performance group (i.e. whether the performance of the hospital in which the participant was based had increased, decreased or stayed the same from the 2015 to 2017 audit). GA started to become familiarised with the interviews’ text by reading (and re-reading) the transcriptions and developed preliminary open codes, which were progressively combined into sub-categories, and then grouped into broader categories. The code structure was iteratively developed as further interviews were added to the dataset. The core categorical scheme that emerged was then applied to all the dataset. During this process the analysis was documented in Memos with explicit links to source text. Links between categories and emerging themes were progressively developed and agreed among authors to check for consistency and validation. Early themes were progressively refined by comparing evidence from data with existing literature. Preliminary results were then summarised in a short report and shared with the NAIF programme team via email. Initial feedbacked helped to refine the study results, which were discussed with the NAIF programme team in a half-day workshop. A revised version of the study report was then shared with the NAIF programme team, who validated findings.

## Results

Results are presented in two macro-areas: (i) report features, local feedback characteristics and actions undertaken following the feedback underling the effective use of NCA feedback to improve local practice; (ii) reported changes in local practice following the NCA feedback.

### Report features, local feedback characteristics and actions undertaken following the feedback

We grouped findings in three broad categories: (1) Report, (2) Feedback, (3) Actions undertaken. (Table 2)

**Table 2:** Report features, local feedback characteristics and actions undertaken following the feedback underling the effective use of NCA feedback to improve local practice.

**a. Report**
**a.1** Benchmarking of performances
**a.2** Simple visual representation
**a.3** Case studies and recommendations
**a.4** Representativeness, credibility and reliability of audit data
**b. Feedback**
**b.1** Different formats (e.g. verbal vs. written)
**b.2** Different professional groups involved (in particular front-line healthcare professionals)
**b.3** Use of graphical tools / visual representation in the presentation and dissemination of feedback
**b.4** Feedback communication simple and straight to the point
**b.5** Encouraging wording and open discussion
**b.6** More frequent or continuous feedback of performance data
**b.7** Feedback reach
**c. Actions undertaken**
**Using data to drive improvement**
**c.1** Use of other relevant data sources
**c.2** Use of continuous monitoring tools (e.g. use of QI tools such as Run Charts)
**Undertaking QI initiatives**
**c.3** Staff engagement and motivation
**c.4** Ownership and clear responsibilities
**c.5** Leadership and communication at different organisational levels (team, ward, Trust)
**c.6** Training
**c.7** Staffing level and turnover/ Resources
**c.8** Organisational culture
**c.9** Senior and operational management support
**c.10** QI skills
**c.11** Supportive QI networks/ collaboratives

**a. Report**.

Participants reported that the presentation of audit results in the NAIF 2017 report allowed them to benchmark local practice compared to the national average and to other hospitals. They also stated that this helped them to identify where improvement was needed and that it served as a trigger to change by providing an opportunity to reflect on current practice and by stimulating a healthy competition with other UK Trusts.

*“I thought the comparison of different trusts was very, very helpful. It makes it a bit more of a competition […] It’s quite a nice healthy competition to improve on your previous results, but also to be better than your neighbours.” H1_Sister*.

*“It was helpful to have a comparison where we put the national average, I think it was a chance for us to sit down and try to reflect what we are doing well and what we are not doing well. It was a trigger to change […] it was a way to reflect on our practice and change something.” H5_Consultant*.

Most participants across the different hospitals liked the summary sheet and the colour coded representation of the audit results as it provided a simple visual representation, which was straight to the point about what the problems were.

*“I liked the way the ratings were […] You know the RAG rating: the red, amber, green rating. The sparkline bit of the documentation, I think, was also quite novel because it gave you an idea of where the gaps were, and it sets about how we understand benchmarking with our regional colleagues and our local colleagues. So that was extremely helpful” H5_Consultant*.

*“I think the summary sheet of the 2017 audit report was useful because it was easy to read, […] was really easy to use and to see at a glance where things could be improved.[…] I suppose I just opened it up and I could find exactly what I wanted to see immediately”. H1_Consultant*.

Key recommendations and case studies were also valued, although interviewees suggested that more examples of good practices put in place by hospitals with high performance would be helpful.

*“It was useful to have the list of recommendations that you can take away when you’re transferring them into the care that you provide.” H1_Matron*.

*“Maybe some examples of people with good practice would be helpful. […] It would have been nice to have some indication of why some centres appeared to have got it all organised better than we managed.” H1_Consultant*.

Some consultants from different hospitals also pointed out the importance of representativeness, credibility and reliability of NAIF report data to their use as a basis for improvement initiatives. Interview data also show that poor representativeness of audit data can compromise staff engagement in future audit activities.

*“I’ve got some doubt about this because it was only a snapshot for only one week and there were only 13 patients, so probably I will have some doubt that it was really representative […] I think probably everyone realise it was a little bit too snapshot!” H5_Consultant*.

*“Well, I felt our data was, I was very confident, because we had a consensus about how we were putting it in and we followed the guidance very closely.[…] I felt we answered it very honestly, I know that, so I’m confident our data was.” H1_Consultant*

**b. Feedback**

For all hospitals, local teams received feedback of audit results. Usually, feedback was obtained at different levels (hospital board, wards, local teams) and by different professional groups (consultants, nurses, physiotherapists, ward managers, matrons, management, those with governance responsibilities, etc.). The feedback was received in multiple formats, either written (e.g. NAIF report, meetings notes, etc.) and verbal (clinical governance meetings, falls committees, ward meetings, etc.).

The NAIF annual report (in particular the summary sheet) was usually used to provide feedback. It was circulated via email and its content (e.g. diagrams, report data) was often pasted in Power Point and presented at meetings.

*“I think the RAG rating is brilliant because they actually give you a colour scheme. […] Giving you numbers and percentages as well is actually quite appropriate. […] It’s also easy to communicate, so when you’re actually putting that on a PowerPoint slide, you’ve got your particular region - so H5 is - and then you can see how you compare to your neighbouring hospitals.” H5_Consultant*.

*“I like the infographics.[…] Things that you can easily print out and use for other people who don’t necessarily have a big interest in it, but it still makes them understand what the audit’s about and what’s been found.” H7_Consultant*.

Participants reported that encouraging wording was used when providing feedback across the different hospitals. Participants reported that ways in which the feedback was provided were appropriate to the effective dissemination of the audit because it was very simple and straight to the point about what the problems were. Moreover, interviewees revealed that the fact that the discussion during the feedback meeting was open and honest was important to the effectiveness of the feedback.

“*We have a very relaxed atmosphere at clinical governance meetings, and everybody, whoever they are, feels that they can speak out […] this helps to identify where change needs to happen*” *H3_Nurse*.

Interviewees also highlighted the importance of continuous monitoring of falls-related indicators and believe that more frequent (or continuous) feedback of NAIF indicators would be beneficial to the prevention of falls.

*“It would be also good to have ongoing information, perhaps quarterly, feedback rather than just yearly.” H2_Assistant Director of Nursing*.

Participants also valued the extension of the audit feedback to front-line healthcare professionals but reported that sometimes it was difficult for clinical staff to dedicate time to these activities due to the pressure of routine work.

“*it would be useful to get a feedback to all the nursing staff, but that is difficult to do, obviously, because of timing. Getting people off the ward, and that kind of stuff*.” *H3_Nurse*.

Finally, some participants highlighted the importance to involve in the audit and feedback process, staff from all over the hospital, not only those working on wards for older people.

“*it [falls prevention]’s always seen as an elderly care problem, even though it’s a hospital-wide issue. I’ve struggled with this every time to get people from other departments and more senior management to be involved in the audit. Everybody feels it’s somebody else’s job*.” *H1_Consultant*.

**c. Actions undertaken**.

**Using data to drive improvement**.

Most interviewees from all hospitals reported that audit data were considered with other relevant data sources before undertaking improvement initiatives. These included mainly falls data not included in the NAIF audit or the Safety Thermometer [69] results. Complementing yearly NAIF audit data with other falls data routinely collected across the hospital helped teams to target improvement initiatives as it allowed them to have a more granular and updated understanding of current practice.

*“All our reported falls data is obviously taken into account, which is where we picked up that falls were happening at certain times of day, or increased falls at certain times of day. So we use our instant reporting data as well.*” *H2_Assistant Director of Nursing*.

Interviewees also find the use of QI tools such as Run Charts useful to monitor the impact of change over time and inform improvement.

*“I think Run Charts are quite important because it gives you continuous data interpretation as you’re going along.” H5_Consultant*.

*“We do use various Run charts and tables which shows the amount of falls that we have every month, and the level of harm from every fall, so we can obviously see if we are improving by doing the work we are at the moment.” H6_Falls Lead Nurse*.

One consultant pointed out the importance to use Run Charts alongside other QI approaches to better understand the causes behind the variations and guide improvement actions.

*“Run charts… It just demonstrates the fluctuations, there’s a good time, there’s a bad time […] rather than anything else more useful […] So that Run chart’s open to different interpretations, and different interpretations will lead to different meaning.[…] So that’s just demonstrate a variation of the same statistic.” H4_Consultant*.

**Undertaking QI initiatives**.

Following the audit feedback, most hospitals undertook QI initiatives, which were usually led locally by 1 or 2 people (falls lead nurse and/ or consultant/NAIF clinical lead). The main reported improvement actions implemented throughout these QI initiatives involved: education and training, updating of the action plan, review of the care plan and of the falls risk assessment booklet, starting internal mini audits, and improving communication to patients and carers. These improvement actions focused on the following main areas: blood pressure monitoring, vision assessment (bedside vision check), medication review, walking aids, continence, dementia, and delirium.

Interviewees from different hospitals reported that engagement of clinical staff and their involvement was key to the identification and implementation of improvement strategies, but at the same time they perceived it as a barrier.

*“I think it [improvement] ‘s centred around engagement of staff, isn’t it? So if the staff can appreciate the importance of falls, they’re going to do something […] So staff engagement is a huge barrier. If they’re well engaged and they understand the process, patient care improves overall.*” *H5_Consultant*.

Most interviewees perceived staffing levels and turnover as a main obstacle to staff engagement and implementation of improvement initiatives following the audit. They also highlighted how organisational culture and senior management support were key to increase staff retention and support effectiveness and sustainability of change initiatives. However, they pointed out how this is hindered by that fact that falls risk prevention activities are often low down in the Trust priorities agenda.

*“Staffing levels is always an issue and continuity of our staff that we have here. […] only 27 per cent of the staff that work here are permanent and we’re having different nurses coming in every day. So any continuity of any initiatives is going to be very difficult to maintain”. H3_Nurse*.

*“This audit is one of the many national audits. I don’t think that’s, in terms of the trust’s priority, that isn’t something that is, anyone pay a lot of attention, other than myself or three or four people in the falls team. In terms of the general, that becomes just one of the many audits that we do in a year.” H1_Sister*.

While participants from five hospitals reported that the organizational culture was supportive and encouraged participation in inpatient fall prevention initiatives, interviewees from two hospitals (H1, H4) reported that audits were mainly owned locally.

*“It’s a very transformational space, supportive, very proactive culture in this hospital[.]. They have an open-door policy where, if you have any significant issues regarding anything that’s highlighted in our Clinical Governance meetings they’re very encouraging in the sense that they want staff to highlight areas where we feel need to be improved and they will help facilitate the improvement […] So that’s one of our mottos, really - collaborative working and working together, facing the future, etc. - as part of our logo of the hospital there*.” *H5_Consultant*.

*“There is the expectation you do the clinical audit but it’s very much left to the local team to decide what’s happening.” H1_Matron*.

*“In terms of quality improvement, I just don’t really see there was a big culture around it.[…] I just felt that it’s a little bit detached with the daily work, if that makes sense.” H4_Consultant*.

Other than drawing the attention to the disconnection between top management and locally owned improvement initiatives, interviewees pointed out that falls’ prevention activities are often limited to older people’s wards and highlighted the need to increase support, communication and involvement of other clinical and administrative hospital departments. One interviewee suggested that a way to do this could be by having a person responsible for inpatient falls prevention activities for each department.

*“I think there should be people nominated from each area, […] a representative from each kind of speciality and not just elderly department.” H1_Matron*.

In general, most interviewees felt that more ownership and clear responsibilities were required and identified inadequate leadership and communication at ward and organisational level (team, ward, trust) as a key barrier to the effective implementation of improvement interventions following the audit.

*“I think somebody who could head the initiative and the audit process and communicate a little bit better would definitely improve awareness and might actually implement change. I think somebody needs to take ownership of the work. I’m not sure who has done that, but they’re not communicating particularly well by the sounds of it.[…] I think somebody needs to take ownership of this for our advice and communicate how we’re doing, and actually get people involved.” H3_Nurse*.

*“We know some of the best work that goes on about preventative work is where you’ve got one individual or a team of individuals who are enthusiasts for the area and keep the pressure on all staff, all healthcare professionals.” H1_Consultant*.

Moreover, interviewees perceived operational management as another factor that could be improved to successfully plan and implement change.

*“I think that could help looking at just basic stuff like the action plans and monitoring, are we meeting the deadlines? If not, why not?” H5_Matron*.

Participants across all hospitals also revealed that training on the audit itself and falls-related technologies would facilitate falls risk reduction improvement efforts. Education on clinical aspects related to falls risk prevention as well as raising awareness on the impact of falls on patient’s quality of life, patient pathways and hospital resources throughout all hospital staff, is perceived as an important support to inpatient falls improvement interventions.

*“I think there’s a real need to get all medical staff looking at falls in terms of medications and understanding blood pressures. So there’s a bit of education needed where we need to empower everybody, not just geriatricians to be thinking about screening people who have fallen, either before hospital or as inpatients.” H1_Consultant*.

*“It’s also making people more aware of the frail elderly, the risk of them falling, the impact of what a fall has on a particular individual, their quality of life, their psychological wellbeing, their health, the level of care and support that they need, the walking aids that may require afterwards; and then the impact it has on the hospital regarding length of stay, the impact on the staff looking after those patients, the impact on the resources used, but also some people after a significant fall won’t be able to get back home.” H5_Consultant*.

Data show that one interviewee attended some training on QI methods and 4/7 study teams did not use structured QI methods like Plan-Do-Study-Act or Process Mapping due to a limited knowledge of these methods or because they felt that these tools weren’t relevant to improve their practice. Some interviewees also reported that the Falls collaboratives (multi-organizational collaborative aimed at supporting local teams to undertake improvement actions to their local context through clinical and QI expert support, guidance in the application of structured QI approaches and methods, peer stimulus and knowledge sharing) supported teams with the use of appropriate QI methods and were key to the success of the QI initiative.

*“I didn’t really consider any [QI] methods on that, I think I probably did it by default. For instance, when you’re doing any change you do things that is a quality improvement tool without realising it.” H5_Matron*.

*“No, we didn’t [do any training on QI methods], although that would have been useful as knowledge of these methods in our team is poor.” H2_Falls Lead Nurse*.

*“They [the use of QI methods] were supported by the falls collaborative to find out if that [*improvement idea*] was working, using the PDSA (Plan-Do-Study-Act) tool.[…] I think some areas were very reluctant to use the tools. They felt that it wasn’t relevant, but once they’d been persuaded in the right direction to use the tool, it was then much easier to see which changes worked, and which didn’t.” H1_Sister*.

Finally, data show how poor engagement and motivation are related in a vicious cycle as interviewees ascribed low engagement and scarce motivation to the frustration of not seeing any change in practice as a result of the audit.

*“I don’t think it’s going to be helpful for me to continue doing this audit. The reason being that 2017 and 2015, the result hasn’t really shown any difference. I didn’t feel that there was lots of changes, so I’m quite happy with what we’ve done with the audit, but I don’t think it is useful to keep repeating the same thing.*“ *H4_Consultant*.

Data also suggest that financial and non-financial incentives might be useful mechanisms to increase motivation. Participants from one hospital with improved NAIF performances (H5), described the successful use of incentive mechanisms related to inpatient falls performances, such as award for posters, publications and presentations, recognition on the monthly hospital news bulletin as well as Clinical Excellence awards and financial incentives for consultants.

### Reported changes in local practice following the NCA feedback

Reported changes in staff behaviour and attitude following the audit included increased awareness, attention and ownership regarding to patient safety aspects.

*“I think there’s more ownership, ward-based […] I think there’s a better understanding, it’s everybody’s problem, but also the importance of why we’re trying to reduce falls, and it’s not just another audit.” H5_Matron*.

*“I think there’s more awareness on the wards and kind of the ward level staff about falls. I think people are more aware of the potential consequences […] I think people talk more about falls and trying to prevent them within the hospital.” H7_Consultant*.

Participants also reported being able to narrow down and focus their efforts toward relevant improvement areas where they were not performing well compared to national benchmark and/or their past performances.

*“So I think it highlighted areas where we weren’t doing too well in, and […] it made us concentrate on seven aspects. People were able to focus on those seven different areas, and that translated into less falls and less harm for the trust and for patients.” H5_Consultant*.

Some interviewees also reported an improved communication and greater involvement of patients and carers in falls prevention because of the audit.

“*We have - we are trying - involvement with patients in preventing falls rather than giving information after a patient has fallen, actually making sure all at-risk patients and relatives have got a leaflet and information that they can use.” H2_Assistant Director of Nursing*.

Finally, some interviewees reported that no change in behaviour was observed following the NAIF audit feedback. For some participants this was due to a perceived lack of representativeness, credibility and reliability of the audit data (and relative feedback), while interviewees from two hospitals reported that difficulty to implement change was due to other competing local priorities.

*“I don’t feel just continuing looking at this is going to bring too much value, but I feel that looking at a different group of patients, say patients admitted like after a week, that would be more helpful to me. […] just repeating the same admissions audit I just felt is not that really going to be useful.” H4_Consultant*.

*“So although we’d put an initial changed programme in, it got impacted on when we went into an electronic patient record. So that’s been affected by a bigger change that happened across the whole organisation.” H1_Matron*.

*“I don’t think the local teams really changed very much as a result of the audit. […] I guess there was other priority from the safety boards, or from the trusts.” H4_Consultant*.

## Discussion

In this study we advance current understanding on how UK NCAs feedback is used locally to improve practice by exploring perspectives of NHS healthcare professionals on a specific national clinical audit, the NAIF audit.

We found that the effective use of a NCAs feedback to improve local practice depends on the way in which data are collected, feedback presented and disseminated as well as on local QI culture, leadership and other organisational mechanisms. NAIF audit feedback enabled participants to identify areas for improvement and to narrow down the scope of QI interventions. Participants reported that feedback also led to an increased awareness and attention to patient safety issues and greater involvement of patients and staff in falls prevention activities.

In accordance with A&F and educational feedback literature, we found that feedback related to reported actions to improve should be perceived as reliable and credible (a.4).[21, 52, 70–72].

Our findings also highlighted the importance of the content of the feedback itself, both in terms of goal setting and correct solution information (e.g. successful case studies) (a.3).[36, 51, 58] We found that benchmarking KPIs with national standards and with other hospitals were valued by audit participants as they helped them to understand where improvement was needed and to target interventions (Table 2, a.1). This finding is consistent with previous feedback literature rooted on organisational psychology and behavioural change literature showing that comparing feedback to goals and standards and redirecting attention towards gaps is key to increase the potential for change following the feedback.[51, 73].

Consistent with previous A&F literature suggesting that feedback should be delivered in both a ‘verbal and written’ format and target people in whom the practice change was desired,[24, 37, 39] in our cases feedback was delivered in different formats and to different professional groups (Table 2, b.1, b.2). While our study participants didn’t express any preference on the way in which the feedback should be delivered (e.g. verbal vs. written), our findings reveal that how feedback data were presented was very important. We found that visual feedback using graphic elements and color-coding was valued by participants and used both in written and verbal feedback as it provided an at glance, straightforward understanding of their current situation and areas for improvement (Table 2, a.2, b.3).

Our study also corroborates A&F literature reporting on the importance of using encouraging wording and of involving clinical staff in the feedback process (Table 2, b.5).[34, 48, 51, 74–76].

In accordance with previous literature, participants reported that more ownership, leadership and time allocated to lead and contribute to the audit itself and to plan and implement QI activities would improve the use of NAIF feedback to drive change in local practice ( Table 2, c.4, c.5, c.7, c.9).[30] Our findings also reveal that front-line staff value the opportunity to have frequent feedback on patient safety issues (Table 2, b.6), and better if it is presented in a visual format (e.g. using Run Charts) (Table 2, c.2). In most cases yearly audit data were used in conjunction with other more frequently collected falls data (Table 2, c.1). The value of interpreting NCA feedback data alongside other routinely collected data and the importance of frequent feedback are mirrored in the wider A&F literature.[2, 36, 37, 39, 48, 51].

Our study shows that the rigorous use of QI approaches (e.g., PDSA cycles, Process Mapping) is still poor mainly due to the lack of knowledge of these techniques by front-line staff (Table 2, c.10). Poor knowledge of QI methods by front-line staff has been widely reported by Improvement Science literature as a major obstacle to QI,[77–82] but has not been a key focus of the narrower A&F literature. In accordance with findings from Improvement Science literature,[83–87] we also found that working with Falls Collaboratives helped to bring in some QI skills and enhanced the use of QI tools to drive improvement (Table 2, c.11).

A great amount of resources, including healthcare professionals’ time, are spent to conduct and feedback on NCAs, with variable evidence on their effectiveness.[2] Yet, previous literature and evidence from this study show that there is still much room for improvement in the effective use of NCA as a QI tool.[2] Therefore, actions need to be taken urgently to optimise their use to deliver change in practice.

While some audit researchers have advocated a standardised approach to audit, based on the definition of clear guidelines on how to conduct a clinical audit (e.g. how the audit is to be undertaken; by whom; when, required steps for audit and feedback processes, etc.),[88–90] other researchers promote a more creative role for audit within QI,[2, 91] which is also reflected in the NICE guidelines.[31] NICE defines A&F as “a quality improvement process that seeks to improve patient care and outcomes through systematic review of care against explicit criteria and the implementation of change”. Differently from narrower definitions of A&F,[24] NICE guidance highlights the importance to integrate A&F within an overall QI framework. According to this perspective, clinical audits should not be seen as isolated interventions. They should instead be embedded in the continuous cycle of improvement and hence be well-thought-out in the strategies and plans for QI of each healthcare organisation.[2] In this way clinical audits become a constituent part of the QI cyclical process, where they are used alongside other QI tools such as Plan-Do-Study-Act,[92] Process Mapping or Real-Time Feedback [88] to drive continuous improvement within healthcare organisations. This approach is perceived to have multiple advantages including a greater clinicians’ engagement due to the fact that these interventions are led by clinicians, fully embedded in their daily clinical practice and use local data for learning on real time feedback with changes to practice being immediate.[88].

However, our findings corroborate previous literature showing that clinical audits often struggle to become a constituent part of routine QI practice within individual organisations and that poor clinicians’ engagement is still a major obstacle to the effective use of NCA to drive change (Table 2, c.3).[2].

Research shows that clinicians’ engagement in improvement activities following clinical audits is difficult to secure when audits and related QI activities are imposed, perceived as additional chore and unnecessary.[91, 93–96] This might happen when a clinical audit is perceived as a ‘political’ tool, a time consuming managerially driven ‘tick box’ exercise used to judge performance with no associated professional reward.[91] Clinicians may perceive it as an additional workload to their routine practice and their engagement and genuine enthusiasm for these activities is reported to be low.[21, 44, 91, 95].

Our findings also confirm previous research showing how low clinicians’ motivation in audit activities is further nurtured by disappointment derived by the failure to deliver the necessary changes following the audit feedback (Table 2, c.3).[91, 95] As a consequence of these failures, clinicians perceive audit as a ‘waste of time’ and this further decreases their commitment in other audits as they feel that their efforts are not worthwhile (Table 2, c.3).[91] In accordance with previous studies [2, 91] participants reported that failure in delivering timely change following audit intervention can be ascribed also to other factors related to the wider organisation, including poor management support and skills as well as lack of resources to support change (Table 2, c.7, c.8, c.9, c.10).

### Implications

Previous research suggests that despite significant financial and infrastructure investment, clinical audits experienced shared challenges, which have also been reflected in the findings of this study. These include poor knowledge of QI approaches and tools as well as a range of attitudinal, behavioural, and organisational barriers to learning and improvement.[97, 98].

A radical change at all levels of health care education and training is required to spread the knowledge of these methods and engage clinicians.[91] In accordance with other researchers,[79, 91, 97, 99] we advocate a more strategic and integrated educational approach embedding audit and QI methods in the undergraduate, postgraduate and life-long learning curricula to all healthcare professions. However, this will require a political will and huge investment of time and resources.[91].

Increased QI training should be accompanied by a QI supportive organisational context. An organisational strategy and local unit climate for quality improvement is an important contextual factor influencing the success of improvement initiatives.[100] A QI oriented organisational culture can help to build a learning environment where people feel free to experiment with change and sometimes fail, to share their experience and to learn from each other. This also potentially stimulates synergistic collaborations with other organisations and networks, which in accordance with previous literature we have found to be beneficial for QI.[78, 83, 85] In addition, appropriate organisational support and managerial skills on multiple levels can help to ensure active involvement of front-line staff by securing time through dedicated resources and to guarantee appropriate management of the initiative through a clear identification of roles and responsibilities.[30].

Finally, although we found that providing a frequent and credible feedback as well as using a visual representation of audit results in feedback activities is important for its effectiveness, research shows that this only rarely happens in practice.

Colquhoun and colleagues in their literature review on A&F interventions in the healthcare setting reported that graphical representation of the data was found in only 36% of the interventions and that rarely feedback was provided on a fast turnaround such as days or weeks.[37] Therefore at organisational level we suggest to put in place monitoring systems providing clinicians with timely and meaningful feedback (ideally continuous) based upon quality indicators relevant to the specific service area and calculated using data perceived as credible.[21, 52] Feedback should be provided using a visual representation and offer recipients more insights on specific areas for improvement to help them focusing their interventions.[24, 36, 101–104] Moreover feedback should be frequent, sustained over time, and tailored to the specific local setting.[20–22, 24, 30, 36, 52, 53, 101–106] This would allow the generation of an evidence base which would motivate, engage and guide clinicians throughout improvement initiatives.

From a research perspective, findings of this study suggest that complementing NCAs and A&F research with theoretical and empirical evidence from the Improvement and Implementation Science literature, could help to investigate the complex mechanisms underpinning the design and delivery of change interventions following NCAs feedback.

### Strengths and limitations

In this study we add to NCAs [107–110] and the wider A&F literature [15, 36, 37, 39] by shedding light on the key factors influencing the effective use of NCAs to improve local practice from the perspective of front-line staff engaged with different roles in the A&F process.

A&F literature provides useful insights on technical aspects of A&F design,[15, 36, 37, 39] while previous studies on NCAs mainly focused on their effectiveness.[11–13, 18, 19].

In this study, the involvement of the perspectives of front-line staff and the broader focus on the practical use of audit feedback to drive improvement, allowed the emergence of aspects which could enhance value of A&F. These have been widely described by the Improvement and Implementation Science literature and include, for example, the improvement of training on QI skills, the value of QI networks, aspects related to the organisational context, such as staffing levels and turnover, organisational culture and management support as well as soft skills, such as the capacity to set up a safe environment for change, leadership and communication skills.[40–46].

Although we studied front-line staff perspectives in the context of a single NCA, we believe that most findings can be generalised to the other UK NCAs (about 50) and NCAs outside the UK. For example, findings related to the NAIF report and to the use of routinely collected data to support the interpretation of audit results might be more specific to the NAIF audit, while other findings related to the implementation of actions following the audit and to feedback dissemination can be generalised to other NCAs. Although the challenges of the UK health system are common to many other countries, the relevance of the findings from this study outside the UK should be contextualised within the specific health system by considering the local QI culture, infrastructure and skills. In terms of generalizability of finding it’s also important to note that at the time interviews were conducted NAIF was a snapshot audit (data collected and fed-back once a year). Since then, NAIF has changed to collect continuous data about fall related inpatient hip fractures. Therefore, some findings from this study might not be still relevant for the NAIF audit or for other continuous NCAs (e.g. the need for continuous feedback of performance data), while new challenges related to the management, presentation and interpretation of complex datasets might have emerged.

There are also other limitations to this study. First, participation in this study was entirely voluntary and many interviewees were people engaged with the NAIF in their organisation. This could cause for selection bias, which we were aware of during the interviews and the subsequent analysis. This has been partially moderated by the inclusion of participants with different professional backgrounds and roles in the NAIF ensuring the identification of a wide range of experiences. However, the identification of the purposive sampling was conducted through snowballing recruitment within each site, potentially causing sampling bias as initial subjects tend to nominate people they know well and have similar traits.

Second, for pragmatic reasons data were collected unevenly across the different hospitals due to issues with the recruitment of busy healthcare professionals. This didn’t allow for comparative analysis between different hospitals or sample subsets (e.g., improvers vs. not improvers) and the dataset wasn’t rich enough to detect the emergence of recurring patterns.

Third, one author (JW) was the NAIF clinical lead. In order to avoid bias due to her role in the NAIF, JW was blinded to the selection of hospitals, and she was only involved in a later stage of data interpretation, when findings were shared with the NAIF programme team. Moreover, researchers followed a reflexive approach throughout data analysis and interpretation by ignoring any personal experiences. Some unavoidable personal assumptions during data categorisation might, however, still exist. Finally, it is solely the experience of the healthcare professionals and their view on the use of NCAs that were in focus. In future research, it could be helpful to engage with hospital managers at different levels and data analysts and gather their views on this as well.

### Further research

Further empirical research on the actual use of NCAs for improvement is required to shed light on key aspects related to the effective use of NCAs to drive improvement. Well-designed process evaluations could help to explore and provide insights into the complex dynamics underlying the variable effectiveness of audit and feedback. Collecting a richer dataset would allow to compare data from different hospitals and/ or groups of hospitals and to identify patterns to explore, for example, the influence of local context (operational practices, organizational culture, etc.) on performance levels. Moreover, this research could be conducted on a broader sample of NCAs with different characteristics to validate and enrich our findings (e.g., different medical specialty, different risk for patient in terms of adverse outcomes, different ways to conduct audit/ present audit results). A review of literature could also be conducted with the objective to synthetise current evidence on NCAs and A&F characteristics which have been found to be relevant to their effectiveness. To balance benefits and pitfalls of different study designs, this review should include studies using a wide range of research methods and approaches, not only RCTs. Findings from the literature review and empirical studies could then been used to outline guidelines (or enriching current guidance – e.g. NICE guidance [31]) to the effective use of NCAs to drive improvement in local practice. This research should be guided by NCAs, A&F, Improvement and Implementation Science literature.

## Conclusions

Although many resources are invested worldwide to conduct NCAs, variable evidence on their effectiveness persists. Front line staff involved in the NAIF 2017 A&F process observed some behavioural changes following the audit, but they also reported that the use of structured QI methods to guide improvement initiatives was poor.

While there are some features of the A&F process that were particularly valued such as the color-coded visual format and the benchmark of performances, there are some aspects that are considered important to the effective use of NCAs feedback to improve local practice which can be improved. In particular, continuous feedback based on data which are perceived as credible, supportive and QI-oriented organisational culture and the introduction of QI training in the curricula of all healthcare professionals could lead to a greater use of NCAs to drive local change.

To make the most of NCAs feedback to improve clinical practice, NCAs should not be seen as isolated interventions, but should be fully embedded and integrated in the QI strategic and operational plans of NHS trusts. This would entail improving long, medium- and short-term planning of infrastructures, resources, activities, roles and training as well as integrating NCAs processes (data collection, feedback, actions following improvement), data and training with the Trust’s broader QI strategy and operations.

Research and insights from Improvement and Implementation Science literature could be used alongside findings from A&F and NCAs literature to explain the complex mechanisms underlying the use of NCAs to improve local practice.

## Electronic supplementary material

Below is the link to the electronic supplementary material.

**Additional file 1:** Consolidated criteria for reporting qualitative studies (COREQ): 32-item checklist

**Additional file 2:** Interview Guide

## Data Availability

The datasets used and analysed during the current study available from the corresponding author on reasonable request.

## List of abbreviations

A&F: Audit and Feedback
COREQ: Consolidated criteria for reporting qualitative research
FFFAP: Falls and Fragility Fracture Audit Programme
FIT: Feedback Intervention Theory
FLS-DB: Fracture Liaison Service Database
HQIP: Healthcare Quality Improvement Partnership
KPIs: Key performance indicators
IRAS: Integrated Research Application System
ICEBeRG: Improved Clinical Effectiveness through Behavioural Research Group
MFRA: multi-factorial falls risk assessment
NAIF: National Audit of Inpatient Falls
NCA: National Clinical Audit
NCAPOP: National Clinical Audit and Patient Outcomes Programme
NHFD: National Hip Fracture Database
NHS: National Health Service
NHSI: National Health Service Improvement
NICE: National Institute for Clinical Excellence
NIHR: National Institute for Health and Care Research
PDSA: Plan-Do-Study-Act
QI: Quality Improvement
RCP: Royal College of Physicians
UK: United Kingdom
USA: United States of America
