# Supplementary material for: How do healthcare providers use national audit data for improvement?
Source: BMC Health Serv Res. 2023 Apr 24;23:393. doi: 10.1186/s12913-023-09334-6 (PMC10123973; doi:10.1186/s12913-023-09334-6)

**Additional file 1: Consolidated criteria for reporting qualitative studies (COREQ): 32-item checklist**


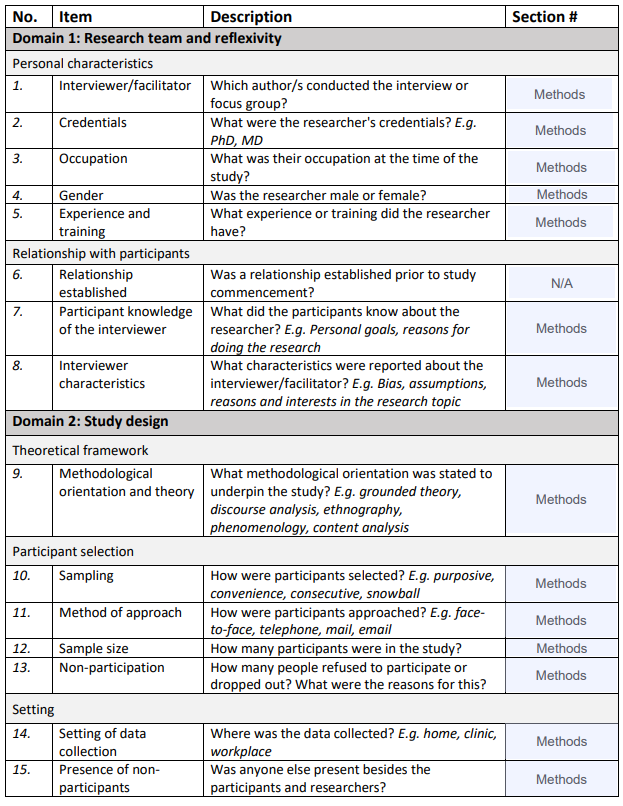


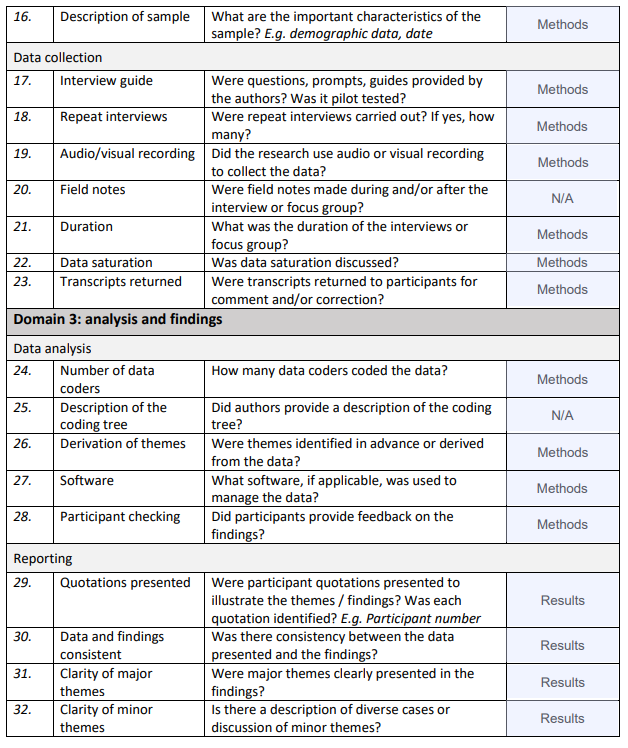

Supplement: Supplementary file 1 — Additional file 1: Consolidated criteria for reporting qualitative studies (COREQ): 32-item checklist [file 12913_2023_9334_MOESM1_ESM.docx]
