# Supplementary material for: How do healthcare providers use national audit data for improvement?
Source: BMC Health Serv Res. 2023 Apr 24;23:393. doi: 10.1186/s12913-023-09334-6 (PMC10123973; doi:10.1186/s12913-023-09334-6)
Supplement: Supplementary file 2 — Additional file 2: Interview Guide [file 12913_2023_9334_MOESM2_ESM.docx]

**Additional file 2**: **Interview Guide**

1. **General Information**

- Please describe your job title and role within the organisation.
- Do you have any previous experience with quality improvement projects?
- Please describe your role in the NAIF audit 2015 and/or 2017?
- Overall do you think that NAIF audit 2015 and/or 2017 were useful to make improvements in your hospital and aid in falls risk reduction?
- What is your experience with the organisational culture of your hospital and unit/ team related to audit and improvement?
- What is your experience with the management style of your hospital and unit/ team related to audit and improvement?

1. **Preparing for audit and data collection**

- Can you tell me what preparation, resources or other infrastructure were put in place to support the effective conduct of the audit?

1. **Feedback process from the RCP - NAIF 2015 and/or 2017 annual report (content, visualization, format, timeliness)**

- Have you seen the RCP - NAIF 2015 and/or 2017 audit report? When? How long ago?
- What did you like about the contents of the audit report and why?
- What was helpful and not so helpful in the audit report and why?
- Were there any aspects you found irrelevant?
- Do you think the indicators used in the audit report were relevant to improve your practice in relation to falls prevention and management?
- What do you think could be improved in the RCP audit report?
- Do you think the visualisation of data in the report was effective to communicate gaps in the service and identify areas for improvement?
- Do you think that data collected were perceived as valid and representative?
- Do you think that continuous feedback of performance data might have made it easier to influence practice in your unit?

1. **Feedback process to local teams**

- Do you remember receiving feedback about your teams’ participation in the 2015 and / or 2017 NAIF audit? If so, when and how? How quickly was the feedback provided after the publication of 2015 and/ or 2017 report? Was the timing of receiving the feedback adequate?
- Did you use the webtool reports prior to the audit report publication to feedback the audit results?
- How were audit results fed back to local teams?
- Do you think that the feedback was perceived as accurate?

1. **Data use and actions**

- How was the NAIF audit data used to help local teams improve quality’? Please give examples.
- Following the feedback, were any internal quality improvement initiatives undertaken?
- Following the audit feedback, were any educational/training activity prompted?
- What actions were undertaken following the audit feedback?
- Have you shared the RCP’s patient report  – *Falls Prevention in Hospital: a Guide for Patients, their Families and Carers* – with patients?
- What changes have been made as a result of 2015 and/ or 2017 audit?
- In your line of work, how often do you receive feedback about prevention activities in your hospital organisation? How?

1. **General – barriers/ enablers**

- What do you think could facilitate falls risk reduction improvement work? And why?
- What do you think could act as barrier to falls preventions improvement work? And why?
- In your opinion, has participation in NAIF audit 2015 and/ or 2017 itself (rather than the data fed back) affected falls prevention work? (if yes) Do you have any evidence to support this opinion?
- Do you remember any relevant differences between the 2015 and 2017 rounds of the audit?
- Are there any additional aspects you wish to raise that have not been covered so far or anything else you would like to feedback?
